# Stop‐Flow Pelvic Chemoperfusion for the Treatment of Malignant Pelvic Bone Tumors: A Preliminary Study

**DOI:** 10.1111/os.12666

**Published:** 2020-04-03

**Authors:** Han Wang, Xiaodong Tang, Lu Xie, Sen Dong, Chen Chen, Wei Guo

**Affiliations:** ^1^ Musculoskeletal Tumor Centre, Peking University People's Hospital Beijing China; ^2^ Department of Radiology Peking University People's Hospital Beijing China

**Keywords:** Chemotherapy, Cisplatin, Pelvic malignancies, Stop‐flow perfusion

## Abstract

**Objective:**

To preliminarily study the efficacy and safety of stop‐flow pelvic chemoperfusion, a novel therapeutic strategy for treating pelvic malignancies.

**Methods:**

Stop‐flow chemoperfusion was performed six times in 5 patients with primary pelvic malignancies. Aortic and vena cave balloons and tourniquets were used to isolate pelvic blood flow from systemic circulation. Cisplatin was then perfused through a transarterial catheter to achieve exposure to a higher drug concentration. Pelvic and peripheral blood samples were collected to determine drug concentration during perfusion. The efficacy of stop‐flow pelvic perfusion was assessed by measuring the change in tumor size, the visual analogue scale, and the tumor necrosis rate after perfusion. Safety was assessed by classifying adverse events according to CTCAE v4.03.

**Results:**

The mean area under the curve (AUC) and maximum drug concentration in the pelvis during perfusion were 246.23 min μg/mL and 17.29 μg/mL, respectively. These measures were significantly higher than the peripheral mean AUC and maximum drug concentration of 52.08 min μg/mL and 5.14 μg/mL, respectively. All 5 patients showed stable disease in response, with changes in tumor size of −4.7%, −5.4%, +4.7%, −8.4%, and 0.0%. Among the 5 patients, 3 (60%) experienced significant pain relief after perfusion. Three patients underwent surgery, with tumor necrosis of 63%, <60%, and 93%. No severe complications were observed in this study.

**Conclusions:**

Stop‐flow pelvic chemoperfusion resulted in exposure to drug higher concentration with fewer serious complications. These preliminary results suggest that further studies are required to comprehensively assess the therapeutic potential of stop‐flow pelvic chemoperfusion in pelvic malignancies.

## Introduction

In comparison to primary malignant bone tumors of the extremities, primary malignant pelvic tumors have poorer responses to neoadjuvant therapies, thereby leading to relatively poor prognosis^1^, ^2^, ^3^, ^4^. Through retrospective analysis of 56 patients with high‐grade pelvic osteosarcoma who received surgery after neoadjuvant chemotherapy at our center^5^, we found only 4 cases that had more than 90% necrosis. Most had 50%–70% necrosis, which demonstrates the inefficacy of intravenous chemotherapy in treating pelvic osteosarcoma. Therefore, novel treatment strategies are required to improve the clinical efficacy of chemotherapy in primary pelvic malignancies.

Intra‐arterial regional chemotherapy was pioneered by Klopp and colleagues in the middle of the last century for locoregional treatment of tumor‐bearing organs^6^. Treatment advances in locoregional chemotherapy were then applied to the treatment of tumors of the lung^7^, liver^8^, ^9^ and the extremities^10^, ^11^, ^12^, ^13^, with good treatment effects. Stop‐flow chemoperfusion is a type of intra‐arterial regional chemotherapy. During stop‐flow perfusion, the blood circulation of the lesion site is limited or isolated, and chemotherapy drugs are directly perfused to the tumor through an arterial catheter to increase drug exposure with the aim of achieving better therapeutic effects and prognosis^14^, ^15^. Reports have shown that stop‐flow perfusion has been applied to tumors in the extremity by using tourniquets and vascular access^16^. Using a meta‐analysis of 1288 patients, Neuwirth *et al*. reported an overall response rate of 73.3% and a complete response rate of 25.8% of isolated limb perfusion and infusion for extremity soft tissue sarcoma^17^.

Due to the complexity of the pelvic vasculature and the difficulty in applying tourniquets, stop‐flow chemoperfusion has rarely been applied to malignant pelvic tumors in the past. Several studies have reported the application of similar techniques to the treatment of pelvic malignancies, such as endometrial cancer and rectal cancer^18^, ^19^, ^20^. However, little research has been done on the application of stop‐flow chemoperfusion to the treatment of primary pelvic bone tumors.

In an attempt to develop a better chemotherapy method, we used aortic and inferior vena cava balloons to achieve stop‐flow pelvic chemoperfusion. This study was designed to investigate: (i) whether stop‐flow pelvic chemoperfusion can achieve a high regional exposure of chemotherapy; (ii) whether tumors show responses to stop‐flow pelvic chemoperfusion; and (iii) whether stop‐flow pelvic chemoperfusion is associated with adverse events.

## Methods

### *Inclusion and Exclusion Criteria*

The inclusion criteria were: (i) patients diagnosed with chemotherapy‐refractory primary pelvic malignancy; (ii) life expectancy >3 months; (iii) no malformation, thrombus, or obvious compression of pelvic vessels and inferior vena cava; (iv) longest tumor diameter >100 mm; and (v) being willing to participate in this study and sign written informed consent.

The exclusion criteria were: (i) aged less than 18 or more than 60 years; (ii) general condition not good enough to receive general anesthesia; (iii) history of thrombosis, ischemic disease, or vascular malformation of the pelvic cavity or lower extremities; and (iv) coagulation insufficiency caused by any reasons.

### *Medical Ethics*

This study was approved by the Ethical Review Committee of our institute. All subjects were required to provide written informed consent to participate in this study. This work was performed in accordance with the Declaration of Helsinki.

### *Perfusion Procedures*

Before perfusion, all subjects underwent contrast‐enhanced CT examination to confirm the location, size, and invasion margins of the tumor and to ensure that perfusion‐related vessels were amenable to interventional procedures. All procedures were performed by senior interventionists, anesthetists, and orthopaedists from our center. The whole process can be divided into the following steps.

#### *Catheter Positioning*

**Figure 1:**
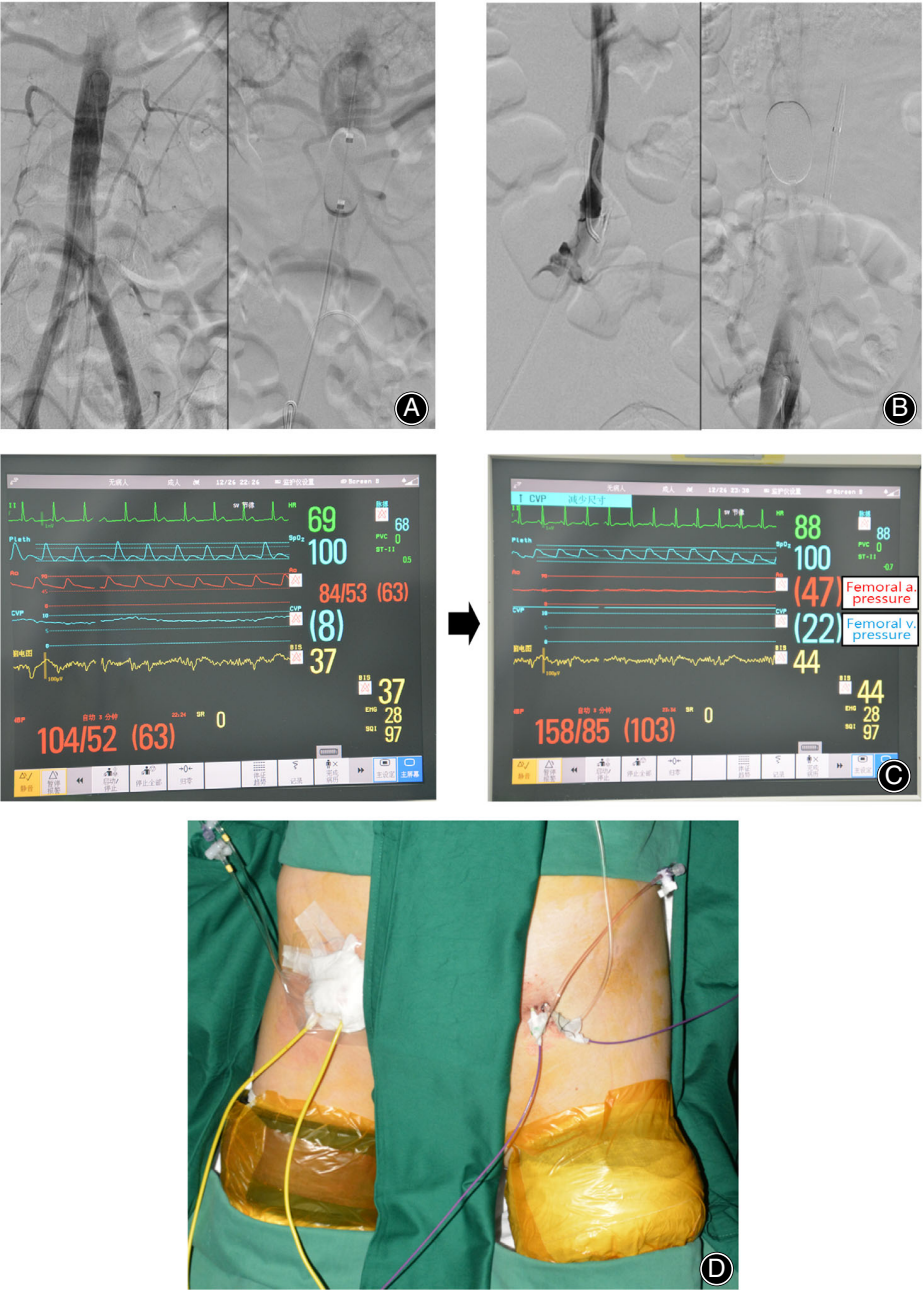
(A) Angiography before (left) and after (right) occlusion showed that the aorta was properly blocked by the balloon and renal arteries were not influenced. (B) Angiography before (left) and after (right) occlusion showed that the inferior vena cava was properly blocked. (C) ECG monitor showed the disappearance of pressure waves from the femoral vessels and obvious decrease and increase in femoral artery and femoral vein, respectively, after occlusion. (D) Intraoperative photo showed that all catheters were ready.

**Figure 2:**
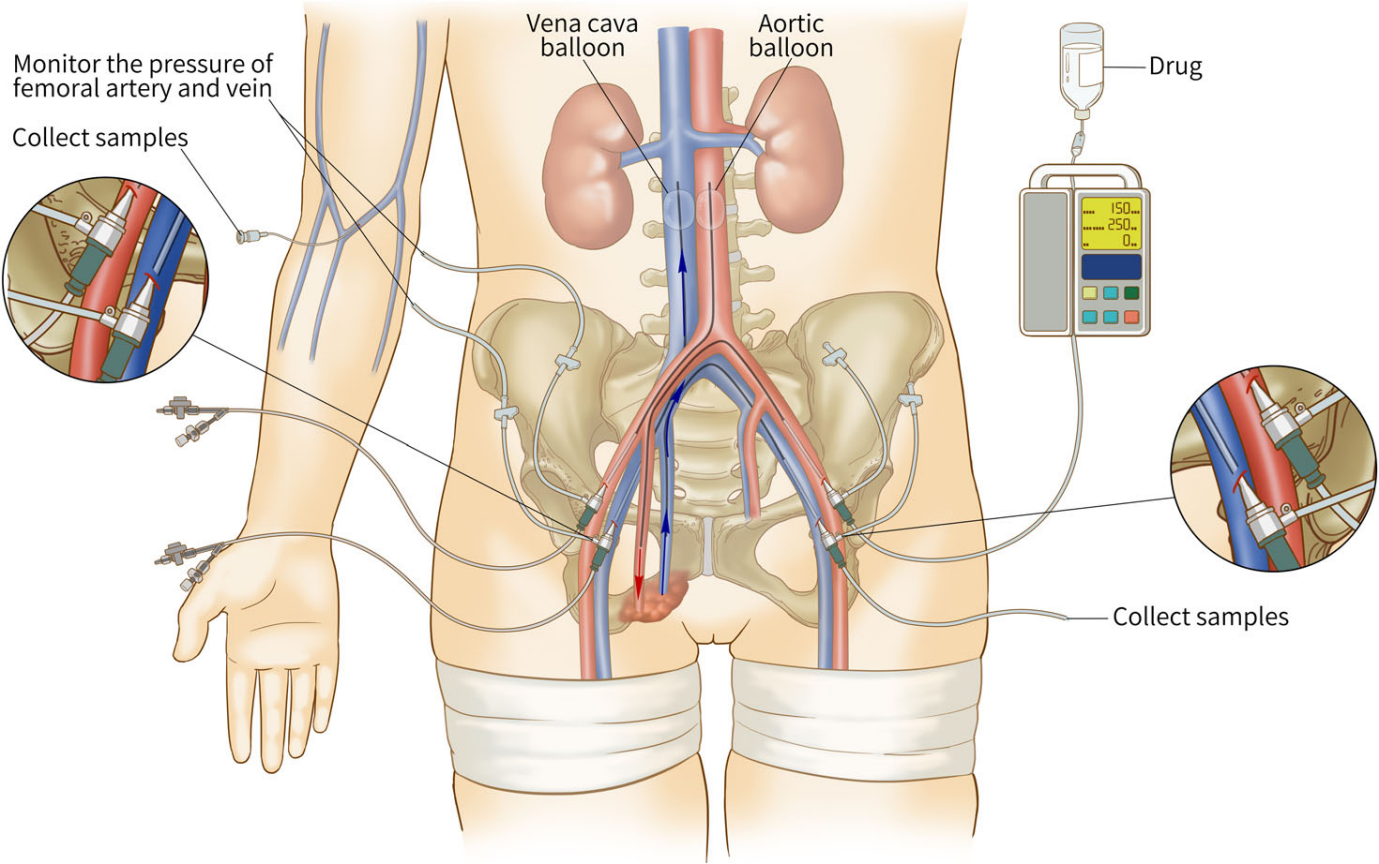
Schema of stop‐flow pelvic chemotherapy. Aortic and vena cave balloons and tourniquets were used to isolate pelvic blood flow from systemic circulation. The drug was perfused through a catheter induced via the femoral artery. Blood pressure was monitored through the catheter sheath in the femoral vessels. The arrows show the flow direction of drugs. Blood samples were collected through the catheter induced via the femoral vein or the peripheral vein of the upper limb.

Under local anesthesia at the puncture sites, bilateral femoral vascular puncture was performed using a Seldinger technique. An 11F catheter sheath (Cordis, Santa Clara, CA, USA) was placed into one femoral artery. A 5F catheter (Cook Medical LLC, Bloomington, IN, USA) was introduced into the T12 level through the sheath under the guidance of fluoroscopy and a guide wire. Arteriography was then performed to observe the position of the bilateral renal arteries and the supply to the tumor. Afterward, an aortic balloon catheter (Cook Medical LLC, Bloomington, IN, USA) was introduced through the 11F sheath into a suitable location between the iliac bifurcation and renal arteries (usually L2/3 level) under the guidance of fluoroscopy and guide wires. A blocking experiment was performed to ensure that no contrast agent could be detected at the proximal end of the balloon and that the renal arteries were not blocked (Fig. 1A). The volume of fluid filled in the balloon was recorded. Through one femoral vein, the vena cava balloon (Cook Medical LLC, Bloomington, IN, USA) was introduced into a suitable location between the iliac bifurcation and renal vessels using a similar guided technique. A blocking experiment was again performed to verify proper occlusion (Fig. 1B). Two other 5F catheters were introduced through the other two femoral vessels. One was placed in the tumor nutrient vessel (usually the internal iliac artery, as determined by previous angiography) to perfuse drugs, and the other was placed in the tumor vein (usually the internal iliac vein, also determined by previous angiography) to collect blood samples during perfusion. Figure 1D shows when all catheters are ready. A schema illustrates the above processes (Fig. 2). Patients were treated with prophylactic low molecular weight heparin to prevent thrombogenesis.

#### *Drug Perfusion*

After satisfactory general anesthesia, pressure monitoring was connected to the femoral vessel sheaths to monitor the pressure of femoral vessels. The balloons were inflated with a recorded volume of normal saline, and tourniquets were inflated to a pressure of 350 mmHg. When the monitor showed disappearance of the pressure wave of the femoral vessels and obvious decrease and increase in femoral artery and femoral vein pressure, respectively, the pelvic blood flow was isolated from systemic circulation, signifying the formation of an isolated perfusion area (Fig. 1C). Chemotherapy was then perfused through the tumor artery catheter using an infusion pump. The infusion speed was set such that all the drug would be delivered within 20 min. During perfusion, pelvic and peripheral blood samples were collected from the tumor vein catheter and upper extremity veins at predetermined timepoints to determine the blood drug concentration. After all drugs were infused, normal circulation was re‐established by slowly deflating the balloons and tourniquets. During the perfusion, femoral vessel pressure and vital signs were closely monitored. When necessary, anesthesiologists gave drugs to maintain the stability of vital signs. All catheters and tourniquets were then removed.

#### *Chemotherapy Drugs*

In this study, we used cisplatin with a dose of 120 mg/m^2^.

### *Sample Collection and Concentration Determination*

The starting point of perfusion was defined as 0 min. Two milliliter blood samples were collected from the tumor vein catheter and upper extremity veins at the timepoints of 0, 5, 10, 15, and 20 min. Two milliliter blood samples were collected from peripheral veins at the timepoints of 25, 60, and 120 min. All samples were collected into heparin tubes.

### *Parameters*

#### *AUC_0–20_* *(Area Under Curve)*

The area under the curve (AUC) is defined as the area under the concentration–time curve and above the X‐axis between the timepoint of 0 and 20 min. The Pt concentration was determined using the inductively coupled plasma mass spectrometry (ICP‐MS) technique. The concentration–time curve was drawn according to the results of drug concentration. AUC_0–20_ was obtained by adding the four trapezoid areas between 0 and 5 min, 5 and 10 min, 10 and 15 min, and 15 and 20 min.

#### *Maximum Concentration*

Maximum concentration is defined as the maximum concentration of Pt detected from the samples collected during the whole chemoperfusion process. The Pt concentration was obtained using the ICP‐MS technique, as mentioned above.

#### *Tumor Response Based on the Change in Tumor Size*

Tumor response is evaluated according to RECIST version 1.1. Briefly, complete response (CR) referred to disappearance of the target lesion; partial response (PR) referred to at least a 30% decrease in the longest diameter of the target lesion; progressive disease (PD) referred to at least a 20% increase in the longest diameter of the target lesion; stable disease (SD) referred to neither sufficient shrinkage to qualify for PR nor increase to qualify for PD. All subjects underwent contrast‐enhanced CT scanning 2 weeks before and 2–3 weeks after perfusion. The longest diameter of target lesions before and after perfusion was measured using the picture archiving and communication system (PACS) at our institute.

#### *Pain Relief*

Pain relief is defined as the change in the degree of pain before and after the perfusion. The degree of pain was evaluated by visual analogue scale (VAS) score. A score of 0 means painless, while a score of 10 means intolerable pain, which seriously affects sleep. The VAS score of all subjects was evaluated 1 day before and 2 days after perfusion.

#### *Necrosis Rate*

He necrosis rate is defined as the percentage of the necrotic tumor in the residual tumor. The necrosis rate was measured routinely by the department of pathology if the patient underwent surgical treatment after chemoperfusion. Briefly, the specimens were dissected along the plane with maximum diameter of tumor and cut into small blocks (approximately 2 cm^2^ for each block). Then the blocks were fixed, decalcified, embedded, sliced, and HE stained. The content and necrosis rate of the tumor of each slice was examined. The necrosis rate of the whole tumor was obtained by calculating the tumor content‐weighed mean of the necrosis rate of each slice. Based on Huvos's criteria^21^, a necrosis rate more than 90% is classified as grade III/IV, indicating a good response to the chemotherapy. Necrosis less than 90% is classified as grade I/II, indicating a poor response to the chemotherapy.

#### *Complications*

Complications are defined as the adverse events occurring within 2 weeks after the perfusion. The complications were graded according to the Common Terminology Criteria for Adverse Events (CTCAE) v4.03. Briefly, grade 1 means asymptomatic or mild consequences, which need no intervention; grade 2 means minimal, local consequences which need noninvasive interventions; grade 3 means severe or medically significant but not immediately life‐threatening consequences; grade 4 means life‐threatening consequences; grade 5 means death related to adverse events.

### *Statistics Analysis*

The significance of the difference in drug concentration between the pelvis and peripheral was determined by Student's *t*‐test using SPSS version 19.0. The level of significance was 0.05. *P*‐values were derived from two‐sided tests.

## Results

### *Demographic Data of the Participants*

Four females and one male were enrolled into this study, with a mean age of 32 ± 3.8 (SD) years. Three of them were diagnosed with pelvic osteosarcoma, and one with mesenchymal chondrosarcoma and one with malignant mesenchymoma. In this article, they were numbered with Arabic numerals. General patient demographic data and baseline characteristics are presented in Table 1. The stop‐flow pelvic chemoperfusion was performed six times in total on these participants (one patient underwent the chemoperfusion twice).

**Table 1:** General data of enrolled patients

Patient no.	Gender	Age	Lesion site	Pathology	Dose of cisplatin
1	Female	33	Right pelvis	Mesenchymal chondrosarcoma	180 mg
2*	Female	28	Right pelvis	Malignant mesenchymoma	180 mg
3	Female	38	Left pelvis	Osteosarcoma	170 mg
4	Male	30	Right pelvis	Osteosarcoma	240 mg
5	Female	32	Left pelvis	Osteosarcoma	190 mg

* This patient underwent the perfusion for two times.

### *Intraoperative Results*

The perfusion of all patients was performed smoothly and the involved procedures were not complicated. What calls for special attention was the changes in vital signs for the dramatic change in the systemic hemodynamics. When the aortic and vena cava balloons were inflated, a temporary increase in heart rate and a decrease in cuff blood pressure occurred. This phenomenon disappeared in a few minutes with or without the application of vasoactive drugs (only administered when deemed necessary by the anesthetists). When the aortic and vena cava balloons were deflated, a temporary increase in heart rate and cuff blood pressure occurred. This phenomenon disappeared in a few minutes with or without the application of vasoactive drugs (only administered when deemed necessary by the anesthetists).

### *Outcome of Pharmacokinetics*

**Figure 3:**
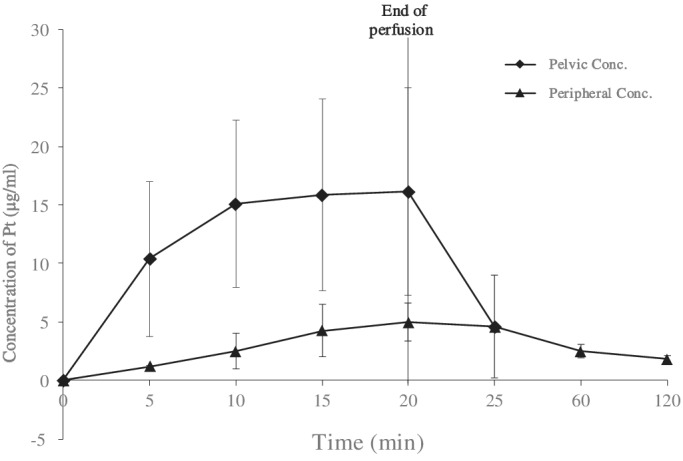
The concentration–time curve during stop‐flow perfusion. The points on the curve are mean concentration at specific timepoints in the pelvis or the peripheral. Error lines are standard deviations.

The concentration–time curve was drawn (Fig. 3), and pharmacokinetic parameters were calculated (Table 2). Figure 3 shows an obvious tendency that the drug concentration in the pelvis was higher than that in the peripheral during perfusion. The mean AUC_0–20_ of the pelvis during perfusion was 246.23 min μg/mL, which was 6.06 times higher than the AUC_0–20_ of the peripheral (52.08 min μg/mL) with statistic difference (*P* = 0.03). The mean maximum drug concentration in the pelvis (17.29 μg/mL) was 4.02 times higher than that of the peripheral (5.14 μg/mL) with statistic difference (*P* = 0.01). An exposure to higher concentration in the pelvis was achieved.

**Table 2:** Pharmacokinetic parameters during perfusion

	Mean (min μg/mL or μg/mL)	Standard deviation	*P*‐value
AUC_0–20_‐pelvis	246.23	131.08	0.03
AUC_0–20_‐peripheral	52.08	26.06	
AUC_0–20_‐ratio	6.06	4.35	
Cmax_0–20_‐pelvis	17.29	7.62	0.01
Cmax_0–20_‐perepheral	5.14	1.84	
Cmax_0–20_‐ratio	4.02	3.00	

### *Outcome of Changes in Tumor Size*

Table 3 shows the changes in the tumor sizes of the patients. We observed slight decreases in tumor size in 4 of 5 patients (80%) after stop‐flow chemoperfusion; 1 patient (20%) showed a slight increase compared with the tumor size before the perfusion. All patients achieved stable disease (SD) based on the criteria mentioned above. The mean change in tumor size was −3.0 mm (SD = 6.8 mm), while one sample *t*‐test with zero showed no significance (*P* = 0.379). The mean change in tumor size of patients with osteosarcoma (patient nos. 3, 4, and 5) was +0.3 mm (SD = 8.0 mm), which showed no significance (*P* = 0.246) compared with the mean change in tumor size (−7.0 mm, SD = 1.4 mm) of patients with non‐osteosarcoma (patients nos. 1 and 2).

**Table 3:** Changes in tumor size after perfusion

Patient no.	Longest diameter before perfusion (mm)	Longest diameter after perfusion (mm)	Change rate	Tumor response
1	170	162	−4.7%	SD
2*	122	116	−5.4%	SD
3	170	178	+4.7%	SD
4	101	93	−8.4%	SD
5	156	155	0.0%	SD

* This table showed the longest tumor diameter before and after the first perfusion of patient 2. SD, stable disease.

### *Outcome of Visual Analogue Scale Score*

Changes in VAS score of all patients are shown in the Table 4. Of the 5 patients, 3 (60%) patients experienced pain relief after perfusion. One patient (20%) showed no pain relief, while one patient (20%) experienced worse pain. The mean change in VAS score was −1.2 (SD = 1.6), while one sample *t‐*test with zero showed no significance (*P* = 0.178).

**Table 4:** VAS before and after perfusion

Patient number	VAS before perfusion	VAS after perfusion
1	2	2
2*	2	0
3	2	3
4	2	0
5	4	1

* This table showed the visual analogue scale (VAS) before and after the first perfusion of patient 2.

### *Outcome of Tumor Necrosis Rate*

Patient nos. 3,4, and 5 underwent surgical treatment 2 weeks after stop‐flow chemoperfusion. Two of them (patient no. 3 and no. 4, 67%) showed Huvos grading I/II necrosis while the other one (patient no. 5, 33%) showed Huvos grading III/IV necrosis.

### *Complications*

Among six perfusions in 5 patients, there was a single occurrence of grade 1 pain at the puncture sites. The pain occurred on the first day after the perfusion and no analgesic was needed. On the third day after the perfusion, the pain was relieved spontaneously. There were also four occurrences of grade 1 nausea, which all occurred on the first day after the perfusion and lasted no more than 3 days. No other antiementics were needed in addition to the routine chemotherapy regimens. No other complications, such as thrombosis, ischemic injury of lower extremities, and renal insufficiency, were observed.

## Discussion

With the development of neoadjuvant chemotherapy, therapies for primary malignant bone tumors of the extremities have significantly improved^1^. However, patients with primary malignant tumors of the pelvis have relatively poor prognosis^2^, ^3^, ^4^. Studies including ours have demonstrated that conventional intravenous chemotherapy is less effective in primary pelvic tumors^5^, which may contribute to the poor prognosis associated with pelvic tumors. Therefore, more effective treatment strategies are needed to improve clinical outcomes for patients with pelvic tumors.

There were several limitations to this study. First, the quantity of enrolled patients was too small to demonstrate significant efficacy of chemoperfusion. Second, other adjuvant therapies were not stopped during this study for ethical consideration, which may have confounded the treatment effects observed in this study. In addition, the pathological disease type of the enrolled subjects may not be sensitive to cisplatin, which may have impacted the resulting therapeutic efficacy of chemoperfusion.

To achieve higher exposure to drugs in this study, we used inflated balloons in the aorta and vena cava and tourniquets to isolate pelvic blood flow from the systemic circulation. A secondary ischemic hypoxic microenvironment was produced, which may have enhanced the antitumor effect of the drugs on the tumors^22^. High doses of chemotherapy were infused into the tumor through a highly selective tumor arterial catheter to achieve a higher dose intensity in the local environment of the target lesions. As this was an exploratory clinical study, the perfusion time was restricted to 20 min to avoid ischemic injuries in normal pelvic organs for ethical considerations.

To explore whether exposure to higher drug concentration in the pelvic region was achieved, we generated a concentration–time curve during perfusion and calculated related pharmacokinetic parameters. The results showed that AUC and maximum drug concentration in the pelvis were significantly higher than those in the peripheral, which is consistent with similar studies^20^, ^23^. The mean AUC_0–20_ and Cmax ratio were 6.06 and 4.02, respectively, which were also consistent with previous reports^20^. We also observed that the concentration in the peripheral slowly rose during perfusion, which might be attributed to drug leakage and establishment of collateral circulation. Some efforts were made to reduce leakage^24^, ^25^. Further studies are required to optimize the technique.

To evaluate tumor response to stop‐flow pelvic chemoperfusion, we measured the changes in tumor sizes and VAS and calculated the necrosis rate after perfusion. All patients showed SD in response to chemoperfusion. It should be noted that all subjects were receiving other adjuvant therapies, including targeted drugs during the course of this study for ethical consideration. This suggests that the combination of perfusion chemotherapy and targeted drugs may be able to control tumor progression. Of the 5 subjects, 3 experienced marked pain relief. There have only been a few reports of tumor response to stop‐flow pelvic perfusion. van IJken *et al*. reported no CR, 1 PR, 10 SD, and 7 PD responses in a study of 13 patients^26^. Bonvalot *et al*. observed 1 CR, 5 PR, 15 SD, and 4 PD in a phase II study of 27 patients^23^. The results of our study were consistent with these previous findings.

Common Terminology Criteria for Adverse Events v4.03 was used to grade all resulting complications of stop‐flow chemoperfusion in this study. No perioperative death occurred. Only a few mild complications were observed. Previous studies reported the application of a similar technique to other pelvic tumors^18^, ^19^, ^20^, ^26^, ^27^. Complications mainly included intervention‐related events and agent‐related toxicity^19^. Aneurysmal dilatations and atheromatous plaques were considered related to vascular complications^28^. In this work, all patients underwent preoperative enhanced CT to ensure vessel integrity, and puncture was carefully performed. We observed no vascular complications. Systemic toxicity is another frequent side effect and the main reason for dose limitation. It is typically related to drug leakage and the kind of drugs used^19^, ^27^. Some investigators have used hemofiltration at the end of perfusion to reduce systemic toxicity^20^, ^29^. However, no significant benefits of hemofiltration were found in some reports^27^, ^30^. In this work, we chose not to perform hemofiltration to allow agents to affect micro‐metastases in other sites because sarcomas easily form small metastases. Hemofiltration‐related complications were thereby avoided and the procedures were greatly simplified. We did not observe any severe systematic toxicity.

In conclusion, the present study was the first, to our knowledge, to report the clinical results of applying stop‐flow perfusion on primary pelvic malignancies. We demonstrated that a higher regional concentration of chemotherapy could be achieved with stop‐flow pelvic perfusion. The safety and efficacy of this procedure were preliminarily assessed in patients with primary pelvic malignancies. Further studies are now required to foster the development of stop‐flow pelvic perfusion as an effective therapeutic strategy for treating primary pelvic malignancies.
